# Expression of costimulatory molecule CD70 is prognostic in small cell lung cancer

**DOI:** 10.1007/s00262-025-04006-2

**Published:** 2025-04-09

**Authors:** David Dora, Zsolt Megyesfalvi, Imre Vörös, Ágnes Paál, Peter Takacs, Daniela Dobos, Bence Lőrincz, Syeda Mahak Zahra Bokhari, Kenan Aloss, Gergely Pallag, Christopher Rivard, Hui Yu, Fred R. Hirsch, Anikó Görbe, Zoltán V. Varga, Zoltan Lohinai, Balazs Dome

**Affiliations:** 1 Department of Anatomy, Histology, and Embryology, Faculty of Medicine, Semmelweis University, Budapest, Hungary; 2 Department of Tumor Biology, National Koranyi Institute of Pulmonology, Budapest, Hungary; 3 Division of Thoracic Surgery, Department of Surgery, Comprehensive Cancer Center, Medical University of Vienna, Waehringer Guertel 18-20, 1090 Vienna, Austria; 4 Department of Thoracic Surgery, Semmelweis University and National Institute of Oncology, Budapest, Hungary; 5 Cardiometabolic and HUN-REN-SU System Pharmacology Research Group, Department of Pharmacology and Pharmacotherapy, Semmelweis University, Budapest, Hungary; 6 Center for Pharmacology and Drug Research and Development, Semmelweis University, Budapest, Hungary; 7 Department of Pharmacology and Pharmacotherapy, HCEMM-SU Cardiometabolic Immunology Research Group, Budapest, Hungary; 8 MTA-SE Momentum Cardio-Oncology and Cardioimmunology Research Group, Budapest, Hungary; 9 Translational Medicine Institute, Semmelweis University, Tűzoltó Utca 37-47, 1094 Budapest, Hungary; 10 Division of Medical Oncology, University of Colorado Anschutz Medical Campus, Aurora, USA; 11 Center for Thoracic Oncology, Tisch Cancer Institute, Mount Sinai Health System, New York, USA; 12 Department of Pharmacology and Pharmacotherapy, Semmelweis University, Budapest, Hungary

**Keywords:** Small-cell lung cancer, CD70, CD27, B-cell, TLS, Tumor microenvironment

## Abstract

**Introduction:**

Small cell lung cancer (SCLC) is a highly aggressive malignancy with poor survival outcomes. The CD70-CD27 axis has been implicated in immune regulation and tumor progression across cancers, but its role in SCLC has not yet been elucidated. This research explores the expression patterns and prognostic significance of CD70 and CD27 in early-stage SCLC.

**Methods:**

In this retrospective study, we analyzed 190 surgically resected SCLC tumor samples using immunohistochemistry (IHC) for CD70 and CD27 expression and RNAscope for CD70 RNA detection. Immune infiltration was assessed using CD45, CD8, and CD20 staining. Quantification of RNAscope signals was performed using QPath software. Kaplan–Meier survival analysis and multivariate Cox regression were used to assess the prognostic impact of CD70, CD27, and immune cell infiltrates on overall survival (OS).

**Results:**

CD70 was expressed in 46% of tumors, primarily within tumor nests, with lower expression in stromal areas. High CD70 expression correlated with significantly decreased OS (*p* = 0.0078, HR: 1.795) without any correlation with CD45 + , CD8 + or CD20 + immune cell infiltrates. CD27 expression was mainly confined to the stroma, and it did not show a significant association with OS (*p* = 0.582). Importantly, high CD27 expression was linked to reduced CD45 + and CD8 + cell densities in the stroma. Both CD70 and CD27 were expressed on CD68 + macrophages, CD27 was expressed on CAFs, and both molecules exhibited a partial coexpression with CD3. Furthermore, patients with high CD20 + B-cell densities or the presence of tertiary lymphoid structures (TLS) had significantly improved OS (*p* = 0.0017, HR: 0.491), suggesting the importance of B-cell-related immune responses in SCLC prognosis.

**Conclusion:**

CD70, B-cell density and the presence of TLSs, but not CD27, emerged as a significant prognostic biomarker for OS in surgically treated SCLC, suggesting its potential as a therapeutic target.

**Supplementary Information:**

The online version contains supplementary material available at 10.1007/s00262-025-04006-2.

## Introduction

Small cell lung cancer (SCLC) is a highly aggressive malignancy. Conventional efforts to repurpose established biomarkers and conduct clinical trials have yielded consecutive failures. Immunotherapy has recently improved the 5-year survival rate of advanced-stage non-small cell lung cancer (NSCLC) patients from 5 to 23.2% [16]. In SCLC, immune checkpoint inhibitor (ICI) combinations have been approved, including the IMpower133 and CASPIAN regimens. However, despite these advancements, the clinical benefit remains incremental, with median overall survival gains of approximately two months, and long-term survival improvements are limited [24], [41]). While historically, the 5-year overall survival (OS) rate for advanced-stage SCLC has remained below 1%, recent data from the IMbrella extension study report a 12% 5-year OS rate for patients receiving carboplatin, etoposide, and atezolizumab [59].

CD70 and CD27 are a ligand-receptor pair, that belong to the tumor necrosis factor (TNF) superfamily [15]. CD27 is found primarily on memory B and T cells, as well as naive T cells, and some subsets of natural killer cells. In physiological conditions, tightly controlled expression of CD70 and CD27 plays a role in co-stimulation in immune response. CD27 activation by CD70 induces a cleavage of the CD27 extracellular domain, and can be also detected as a fragment (sCD27) in bodily fluids [27, 28], [48]. However, its role in the tumor microenvironment (TME) is far more multifaceted [15], [51]. Dysregulation of the CD70-CD27 axis within the tumor and its microenvironment is associated with tumor progression and immunosuppression [15]. Interestingly, abnormally high levels of sCD27 have been found to be present in patients presenting with a hematological malignancy, which suggests the involvement of the CD70-CD27 axis [54], [56]; [20], [48]). Tumor cells regulate CD27 expression in the TME by expressing CD70, which promotes immune escape [34]. Furthermore, CD27 signaling increases regulatory T cell (Treg cell)-infiltration promoting tumor growth [9].

The CD70-CD27 axis plays a key role in hematopoiesis. CD27 is present on murine progenitor cells [37] and CD70 inhibits leukocyte differentiation via CD27 activation [37]. Targeting this axis has been explored in hematological neoplasms, including Hodgkin lymphoma, diffuse large B-cell lymphoma [4, 43], and chronic- [49] and acute myeloid leukemia ([47]; [50]). In multiple myeloma, CD27 expression decreases with disease progression [22]. However, in the last decade, the therapeutic prospect of the CD70-CD27 axis has gained more significance in solid tumors as well. Its indubious role has been elicited in renal cell carcinoma ([10]; [29]), ovarian carcinoma [35], pleural mesothelioma [25], breast cancer [42], malignant melanoma [44], and NSCLC [27, 28]. In glioblastoma multiforme (GBM), CD70 plays a key role in recurrent GBM cell aggressiveness and maintenance. In SCLC, it was reported that immune cell infiltration and expression of immune checkpoints and other immunostimulatory molecules, including IDO, PVR or STING are increased in the neuroendocrine-low (or variant) subtype [11], the latter identified as a positive prognosticator along with CD8 + immune cell infiltration [13]. Furthermore, RNAseq data on a subcohort of *n* = 30 early-stage SCLC patients showed that CD70 is overexpressed in the more immunogenic NE-low subtype of SCLC [12]. To date, no data is available on the tissue expression pattern and prognostic role of the CD70/CD27 axis in SCLC.

In the current study, we analyzed the RNA and protein expression of CD70, using RNAscope and immunofluorescence (IF) and the protein expression of its receptor, CD27, including its correlation with CD45 + CD8 + immune cell infiltration previously reported in [13] on a large, real-word cohort of early-stage SCLC patients. Furthermore, we quantified the tissue expression of CD70’s receptor, CD27 in the TME and its colocalizing expression signals with tumor- stromal and immune cells. Because the CD70/CD27 axis is heavily involved in B-cell biology in physiological conditions and in multiple pathologies, we also aimed to quantify B-cell densities in SCLC tumors, that has not been reported so far in any real-word cohort, including its correlation with CD70 and CD27 expression in situ. In the clinical setting, we showed that increased CD70 RNA expression significantly deteriorates overall survival (OS) in early-stage SCLC patients, making it a suitable target for future biomarker studies and clinical trials.

## Materials and methods

### Ethical statement

The study was carried out in accordance with the Helsinki Declaration guidelines of the World Medical Association Hungarian scientific ethics committee (ETTTUKEB-7214–1/2016/EKU). Due to the retrospective nature of the study, the requirement for individual informed consent was waived. Following the acquisition of clinical data, the patients' identities were anonymized, ensuring that the patients cannot be identified either directly or indirectly.

### Study population

In this retrospective study, 190 surgically-resected and histologically confirmed consecutive SCLC patients were included between 1975 and 2013 at the National Koranyi Institute of Pulmonology, Budapest, Hungary. The study and all treatments were conducted based on the institutional guidelines. The date of the last follow-up included in this analysis was April 2022. Clinicopathological data included age, gender, disease stage and details concerning adjuvant chemotherapy and overall survival (OS). Table 1 shows clinicopathological characteristics for patients with available clinical data according to CD70 expression (high vs low).

**Table 1:** Clinicopathological characteristics of the cohort with available clinical data according to CD70 expression (high vs low)

Clinical characteristic	CD70-High	CD70-Low	*p*-value*
Stage I/II	28 (73.7%)	21 (63.6%)	0.443
Stage III	10 (26.3%)	12 (36.4%)	
Gender (Female)	31 (81.6%)	25 (75.8%)	0.574
Gender (Male)	7 (18.4%)	8 (24.2%)	
Adjuvant CT (Yes)	23 (60.5%)	18 (54.5%)	0.637
Adjuvant CT (No)	15 (39.5%)	15 (45.5%)	
Overall survival (median, months)	12.6	33.3	0.02
Age (median, years)	57.0	58.0	0.583

*P* values for Mann Whitney U test in case of continuous variables and Chi-squared test in case of categorical varibles. Percentages refer to the percent of total patients with available clinical data stained for CD70

### Tissue processing

Following surgery, SCLC tumor samples underwent fixation and were subsequently processed into paraffin blocks. The construction of tissue microarrays (TMAs) from formalin-fixed paraffin-embedded (FFPE) blocks was conducted in accordance with established methods [3]. In brief, 4-micron sections were meticulously sliced from each tissue block using an HM-315 microtome (Microm) and deposited onto charged glass slides (Colorfrost Plus, #22–230-890, Fisher). These slides were subjected to hematoxylin and eosin (H&E) staining using an automated Tissue-Tek Prisma staining platform (Sukura). An experienced pathologist, certified in thoracic oncology, examined the H&E-stained slides to identify the tumor area, demarcating its boundaries. Two 1-mm tissue punches were extracted from each donor tissue block and precisely positioned into a recipient paraffin block, arranged in an encoded array format using an MP10 1.0-mm tissue punch on a manual TMA instrument (Beecher Instruments).

### Immunohistochemistry and immunofluorescence

For the immunohistochemistry (IHC), five-micron-thick sections were prepared. The staining was carried out on a Leica Bond RX autostainer with rabbit monoclonal antibodies against CD20 (#48,750) from Cell Signaling and CD27 (#131,254) from abcam, diluted to 1:200 following the protocols established by [13]. Briefly, after primary antibody binding sites visualization, slides underwent staining with the Bond Polymer Refine Detection kit (#DS9800) following Leica IHC Protocol F. Epitope retrieval 1 (low pH) was conducted for 20 min. Hematoxylin was used for counterstaining. For dual Immunofluorescence (IF) stainings, we used antibodies against CD70 (#300,083), CD27 (#131,254), CD68 (#201,340), and CD3 (#201,340) from abcam and CD20 (#14–0202-82) and Vimentin (VIM, #MA5-11,883) from Invitrogen, each at a 1:200 dilution in 1% PBS/BSA. Dual immunofluorescence (IF) employed Alexa IgG anti-rabbit A488 and IgG anti-mouse A546 fluorescent secondary antibodies from Invitrogen for detecting signals from CD70, CD27, CD3, CD20, CD68, and VIM antibodies. Autofluorescence on FFPE samples was eliminated with TrueView® Autofluorescence Quencher (Vector). Human tonsil and lung adenocarcinoma tissues served as positive controls for protein expression assessment.

### RNAscope

The RNAScope® ISH assay was executed on tissue microarrays (TMAs) employing the RNAScope® Multiplex Fluorescent Kit v2 in accordance with the manufacturer's protocols (Advanced Cell Diagnostics Pharma Assay Services, Newark, CA, USA) and as previously described [14]. Briefly, 4 µm sections of formalin-fixed paraffin-embedded TMA were pretreated with antigen-retrieval buffer, heat, and protease. Subsequently, hybridization was performed with the following target oligo probes: 3plex-Hs-Positive Control Probe (ACDBio, cat: 320,861), 3plex-Hs-Negative Control Probe (ACDBio, cat: 320,871), and Hs-CD70 (ACDBio, Cat No. 419331). Preamplifier, amplifier, and AMP-labeled oligo probes were then sequentially hybridized, followed by the development of fluorogenic precipitates (Cy3, red). Each sample underwent quality control to assess RNA integrity using a positive control probe specific to housekeeping genes, and a negative control probe set was employed to evaluate background fluorescence. Nuclei were counterstained using 4',6-diamidino-2-phenylindole (DAPI).

### Scoring the CD70 RNAscope signal with QPath

To quantitatively assess RNA expression, we employed the Qpath software package, following established methods and equations to quantify the dotted fluorescent signals in our regions of interest (ROIs) ([2]; [14]. Briefly, we employed DAPI nuclear staining to delineate individual cell regions by assigning each cell's radius, designating each cell as a region of interest (ROI). Subsequently, we quantified the number of dots within each ROI, following established procedures. An integrated expression score within the [0–3] range was calculated for all tumor cores, based on the average density of dots and raw intensity data. The determination of cut-offs was performed using the k-means clustering method. For all tissue microarrays (TMAs), two cores per patient were included, and an average CD70 expression score was computed for each patient. The median value for aggregate CD70 scores was 2.0. To ensure balanced classification of CD70 “high vs “low” tumors, we decided to include tumors scoring 2.0 to the CD70 “high group. All intensity measurements were conducted utilizing an LSM780 Zeiss confocal microscope and Zen Blue software package.

### Scoring of CD20 and CD27 expression

Images of TMA sections were captured for scoring and cell counting using a BX53 upright Olympus microscope equipped with a DP74 color CMOS camera at 10 × magnification in 20MP resolution, and representative images from tumor tissues were captured at 20 × magnification. For all TMAs, two cores per patient were included. From all TMA blocks, two separate four-micron-thick sections, spaced at least 100 μm apart in the Z-axis, were quantified. The ImageJ software package was used to perform cell counting, where microscopical images of sectioned and stained TMA cores were imported and followed by conversion to an 8-bit grayscale format to enhance contrast. The "Threshold" function was used to define and isolate the cells from the background, with adjustments averaged using 100 images manually counted to yield an accuracy of ± 5%. Adjustments were made to ensure proper identification of individual cells. Subsequently, the "Analyze Particles" feature was utilized to count the cells, with size and circularity parameters set to exclude debris or cell clusters, using the above-mentioned averaging. A macro was then created and applied to all samples. The results were then recorded as the total number of cells per BLANK.

### Datasets

To assess the single-cell expression pattern of the CD70/CD27 axis in the healthy lung, we used the Human Protein Atlas (HPA, http://www.proteinatlas.org/, [53]). To evaluate CD70 and CD27 expression in the TME of lung cancer, Broad Institute’s Single Cell Portal was used compiling data from Zilionis et al. [58], where a comprehensive scRNAseq profiling of myeloid and lymphoid TME cell populations was carried out on *n* = 7 NSCLC patient samples. RNA-seq data are reported as average fragments per kilobase of exon per million reads mapped (FPKM) for both datasets used. Single cell expression levels of major cell types and the proprietary LM22 immune cell set was used to assess the expression pattern of the axis in human lung cancers. LM22 is a signature matrix file distinguishing 22 mature human hematopoietic populations isolated from peripheral blood or in vitro culture conditions [7].

### Statistical analyses

Normality was evaluated using the Shapiro–Wilk test and nonparametric Mann–Whitney U-test was used for cell density comparisons. Comparison of TLS presence with CD70 and CD27 phenotype was performed using the chi-squared test. Survival analysis was performed with Kaplan–Meier curves and a comparison of survival curves with the log-rank test. Cox-proportional hazard regression was used to screen for significant predictor variables. The analysis was two-sided with a level of significance of α = 0.05. To assess the goodness of fit of our multivariate model, Harrel’s C-index (> 0.7) was calculated. Violin charts were visualized using GraphPad Prism 8.0 and KM-curves were generated with the MedCalc 14 statistical software package.

## Results

A total of *n* = 190 patients were included in our study with resected SCLC of whom *n* = 167 patients had scoreable CD70 RNAscope stainings and *n* = 182 CD27 stainings. CD20 staining was available for *n* = 173 patients. Clinicopathological data were available for *n* = 88 patients (*n* = 68 early-stage, *n* = 20 advanced stage (III/A)).

### The CD70/CD27 axis is expressed in healthy lung and lung cancer

According to single-cell data retrieved from the Human Protein Atlas (HPA), costimulatory molecules CD70 and CD28 are both expressed in the healthy lung. CD70 is mainly expressed in B-cells, and in plasma cells in line with the molecule's biology in healthy hosts, with considerable expression in Langerhans- and T-cells, basal respiratory cells and fibroblasts as well (SFig 1A). CD27 is predominantly expressed in plasma cells, but also in B-cells, with notable expression levels in T-cells and erythroid populations (SFig 1B) according to HPA data, suggesting the lympho-myeloid dominance of the axis in physiological conditions. In the healthy lung, based on single-cell data, CD70 is expressed in B-cells and two distinct T-cell populations, with a minor presence on macrophages. Club cells and endothelial cell populations also show traces of CD70 expression (SFig 1C). CD27 is predominantly expressed in B- and T-cells with a small addition from different macrophage populations (SFig 1D).

**Fig. 1:**
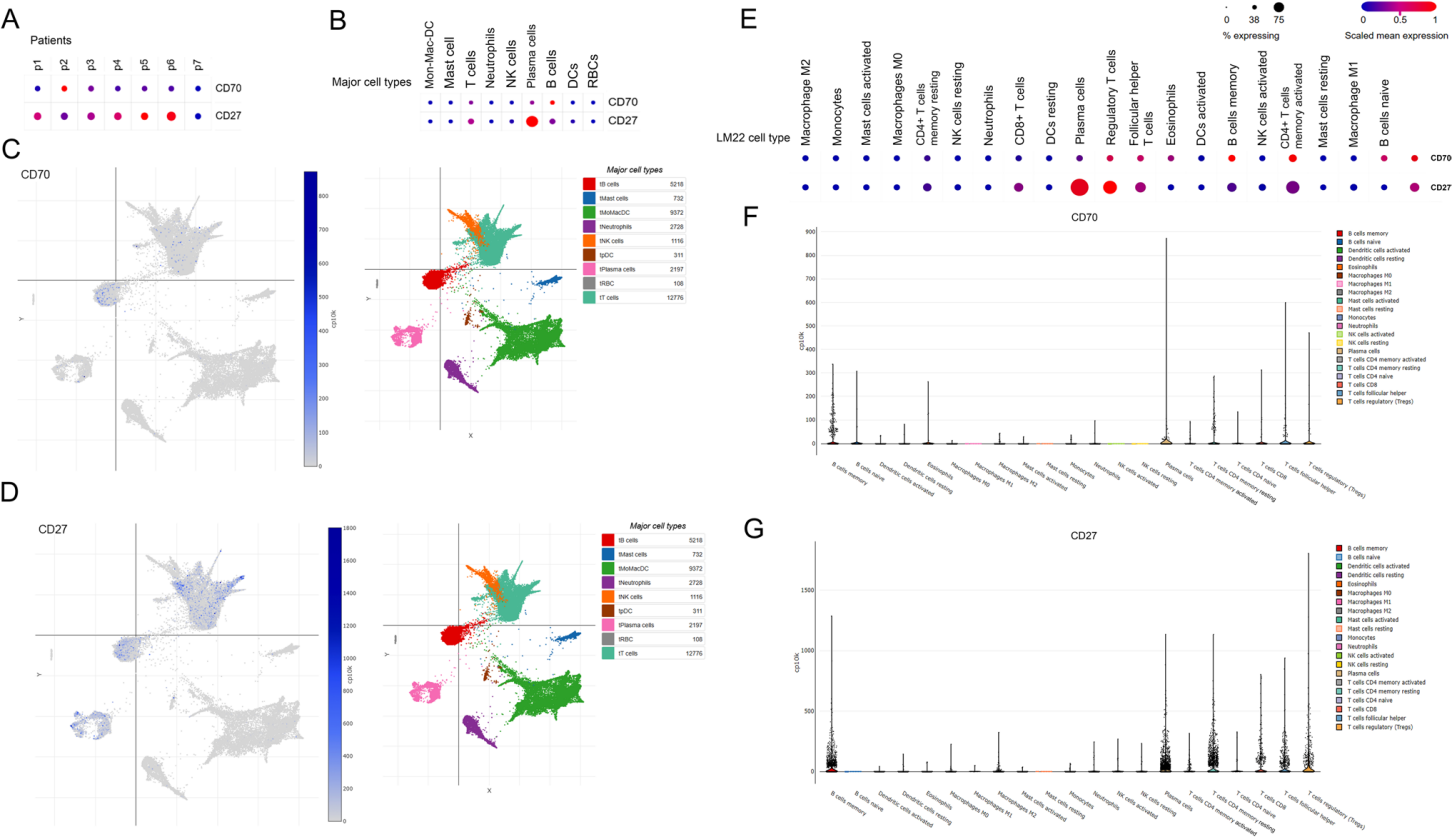
Expression of the CD70-CD27 axis in NSCLC. Single-cell data from the SingleCell portal show expression means and percentage of expressing cells in dot plots comparing CD70 and CD27, according to included patients (**A**) and major cell types (**B**). UMAP plots show the number and cluster of cells expressing CD70 (**C**) and CD27 (**D**). CD70 is expressed in B-cells, CD4 + T-cells (naive, memory), T-regs, and eosinophils within the NSCLC tumor microenvironment according to the LM22 signature matrix file (**E**, **F**). CD27 is primarily expressed in plasma cells, T-regs, follicular helper T-cells, and other CD4 + T-cell populations based on the same dataset (**E**, **G**). Row minimums and maximums (RNA expression, Log(CP10k + 1) normalized counts matrix (genes by cells)) in panel A are color coded. Size of corresponding circles correlates with the percentage of expressing cells for every cell type in dot plots

Data retrieved from the SingleCell (SC) portal suggest that CD70 and CD27 are also expressed in the TME of NSCLC. 1 from 7 patients showed high-, 3 from 7 showed modest CD70 expression when observing all cell types (Fig. 1A). In contrast 6 from 7 patients exhibited moderate-to-high CD27 expression levels (Fig. 1B). When observing single-cell expression levels specifically for major cell types and LM22 cell populations, it is shown that CD70 is mostly expressed in B-cell populations and plasma cells, and in CD4 + T-cells (both naive and memory), regulatory T-cells (T-regs) and eosinophils to a smaller extent (Fig. 1C, E, and F). CD27 shows the most prominent expression in plasma cells, where a considerable percentage of the cell type’s cells express the protein, but also T-regs, follicular helper T-cells and other CD4 + T-cell populations show notable expression of CD27 (Fig. 1D,E and G).

### Expression of CD70 and CD27 in SCLC

**Fig. 2:**
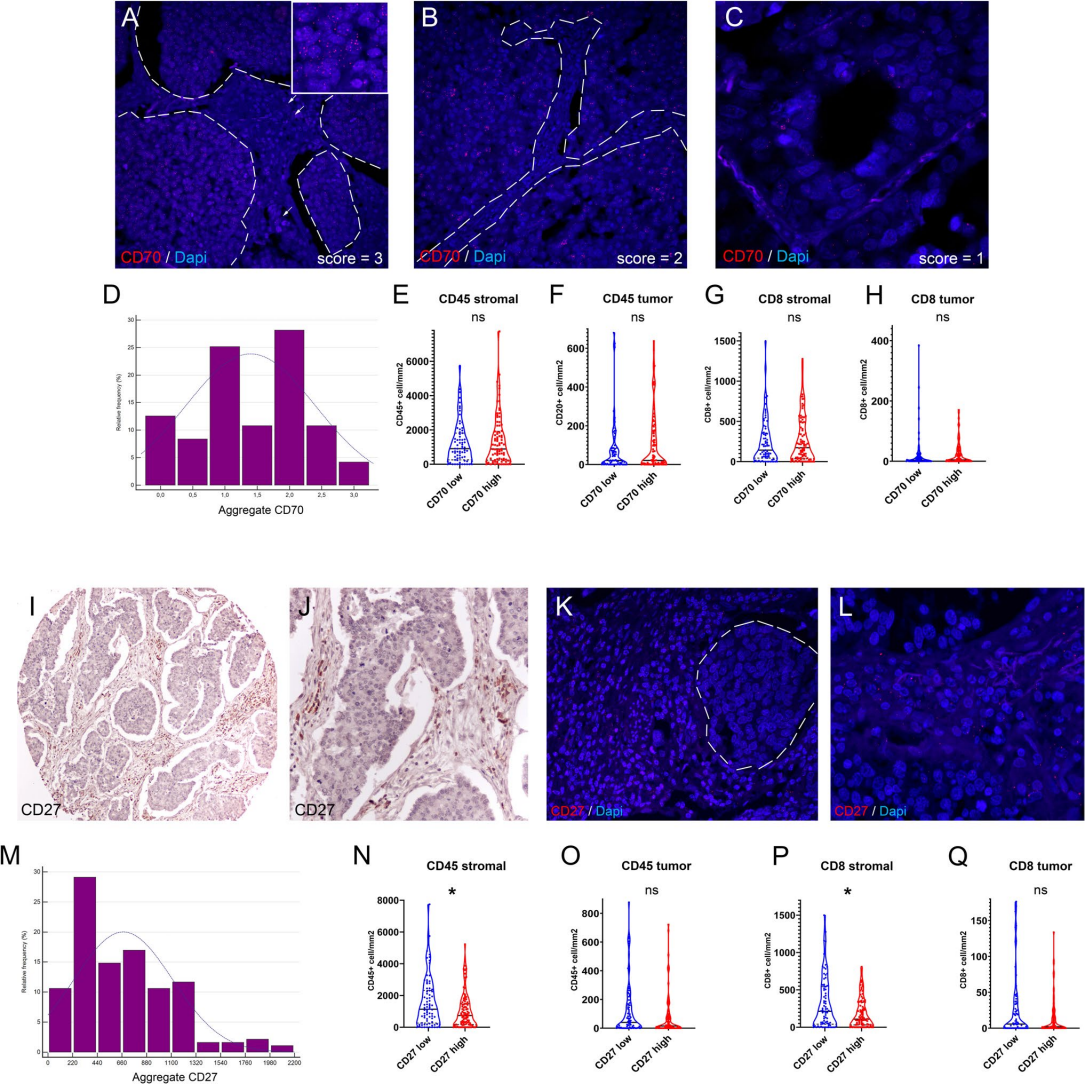
Expression of CD70 and CD27 in SCLC. RNAscope assays revealed CD70 expression predominantly in tumor nests (A,B, dashed line delimited areas) and scattered in stroma (**A**, arrows), with most tumors scoring 1.0–2.0 (**B**,**C**). Tumors with CD70 scores of 2.0–3.0 were classified as “high,” and 0–1.5 as “low.” Histogram shows distribution of CD70 scores (D). CD70 expression was not significantly associated with CD45 + or CD8 + cell densities in stroma or tumor nests (**E**–**H**). CD27 was primarily expressed in stroma (**I**-**L**). Distribution of CD27 aggregate scores through tumor samples is shown in panel **M**. High CD27 + cellular density was linked to reduced CD45 + and CD8 + cell densities (**N**,**P**), but not in tumor nests (**O**,**Q**). Metric data are shown as medians and 95% CI on violin plots. Statistical significance *P < 0.05; **P < 0.01, ***P < .001. All *p* values were two-sided

RNA Expression scores were established for each patient based on RNAscope assays and an area-adjusted quantitative scoring system using QPath. Panels in Fig. 2A-C show representative images of strong (score = 3), moderate (score = 2) and low (score = 1) expression levels for CD70. CD70 RNA signals were predominantly located in tumor nests (Fig. 2A,B *dashed line areas*), but also scattered in stroma (Fig. 2A *arrows*). Histograms of CD70 score distribution through patients show similar to normal distribution with most tumors scoring 1.0–2.0 (Fig. 2D). All tumors with aggregate values of 2.0–3.0 were classified as CD70 “high”, and with values of 0–1.5 were classified as CD70 “low.” Next, we compared CD45 + immune cell- and CD8 + T-cell densities separately in the stroma and tumor nest compartments according to CD70 expression using data published earlier in the same cohort [13]. It is shown that CD70 RNA expression is not associated significantly with immune cell infiltrates neither in the stroma (Fig. 2E,G), nor in the tumor nest compartments (Fig. 2F,H). Thus, high CD70 expression does not come with categorically higher- or lower immune cell densities.

Expression of CD27 was evaluated on the same cohort using an IHC-based quantitative scoring system described earlier [11],2022). CD27 is expressed predominantly in the stroma compartment (Fig. 2I,J), which was confirmed using RNAscope as well (Fig. 2K,L) Histogram shows the distribution of tumors according to CD27 + cellular densities (Fig. 2M). In contrast with CD70, it is shown that high CD27 expression was associated with decreased CD45 + (Fig. 2N) and CD8 + cellular densities (Fig. 2P), but only in the stroma compartment, not in tumor nests (Fig. 2O,Q). Neither CD70 expression (rs = -0.0421, *p* = 0,713), nor CD27 + cellular density (rs = 0.0165, *p* = 0.881) show significant correlation with tumor stage, suggesting an expression pattern inherent to the original tumor phenotype.

### B-cells and tertiary lymphatic structures (TLSs) in SCLC

**Fig. 3:**
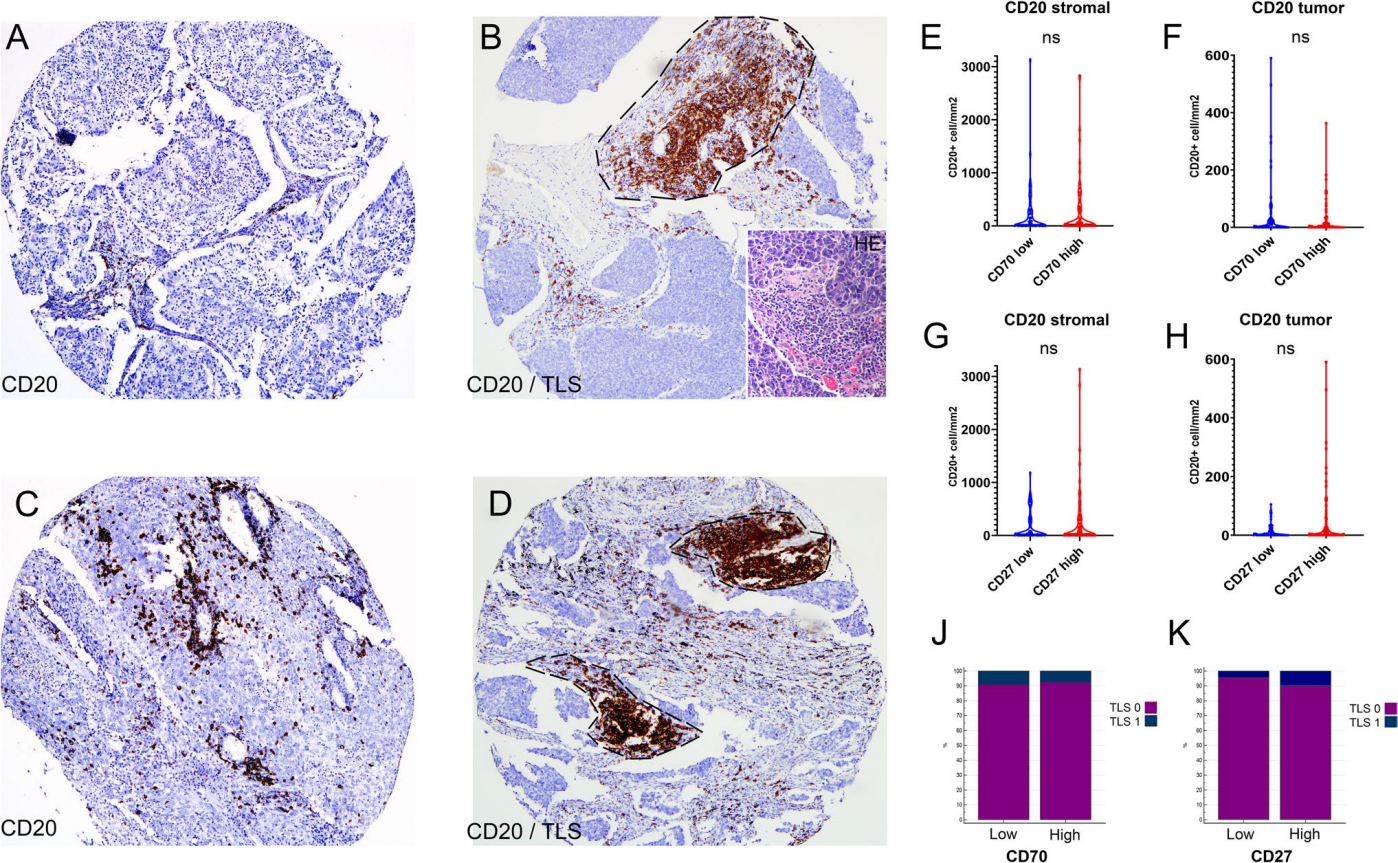
B-cells in SCLC in the context of the CD70-CD27 axis. CD20 + B-cell densities were evaluated in the SCLC cohort using the same scoring method as in the case of CD27. B-cells are primarily located in stromal bands, with occasional intratumoral presence and TLS formation (**A**, **B**). Multiple tumors showed signs of formation of tertiary lymphatic structures (TLSs) made up from congregations of CD20 + B-cells (**C**,**D**, dashed line delimited area). No significant association was found between CD70/CD27 expression and B-cell densities or TLS presence (**E**–**H**, **J**, **K**). Metric data are shown as medians and 95% CI on violin plots. Statistical significance *P < 0.05; **P < 0.01, ***P < .001. All p values were two-sided

We also evaluated CD20 + B-cell densities in our SCLC cohort. Representative images show tumor TMAs with low (Fig. 3A) and relatively high (Fig. 3B) B-cell densities. As expected, B-cells are mostly located in stromal bands, but also occur intratumorally and occasionally create congregations known as TLSs. We identified a total of *n* = 14 patients (8%) with TLS present in their tumors, as shown in stainings on Fig. 3C and D. When comparing CD20 + B-cell densities with the expression of CD70 and CD27, we found no significant association between the immunomodulatory molecules and B-cell densities (Fig. 3E-H) neither in stroma nor inside tumor nests. We came to the similar conclusion, when assessing the presence of TLSs along with CD70- and CD27 expression in the same cohort (Fig. 3J,K).

### Expression of the CD70/CD27 axis in SCLC TME

**Fig. 4:**
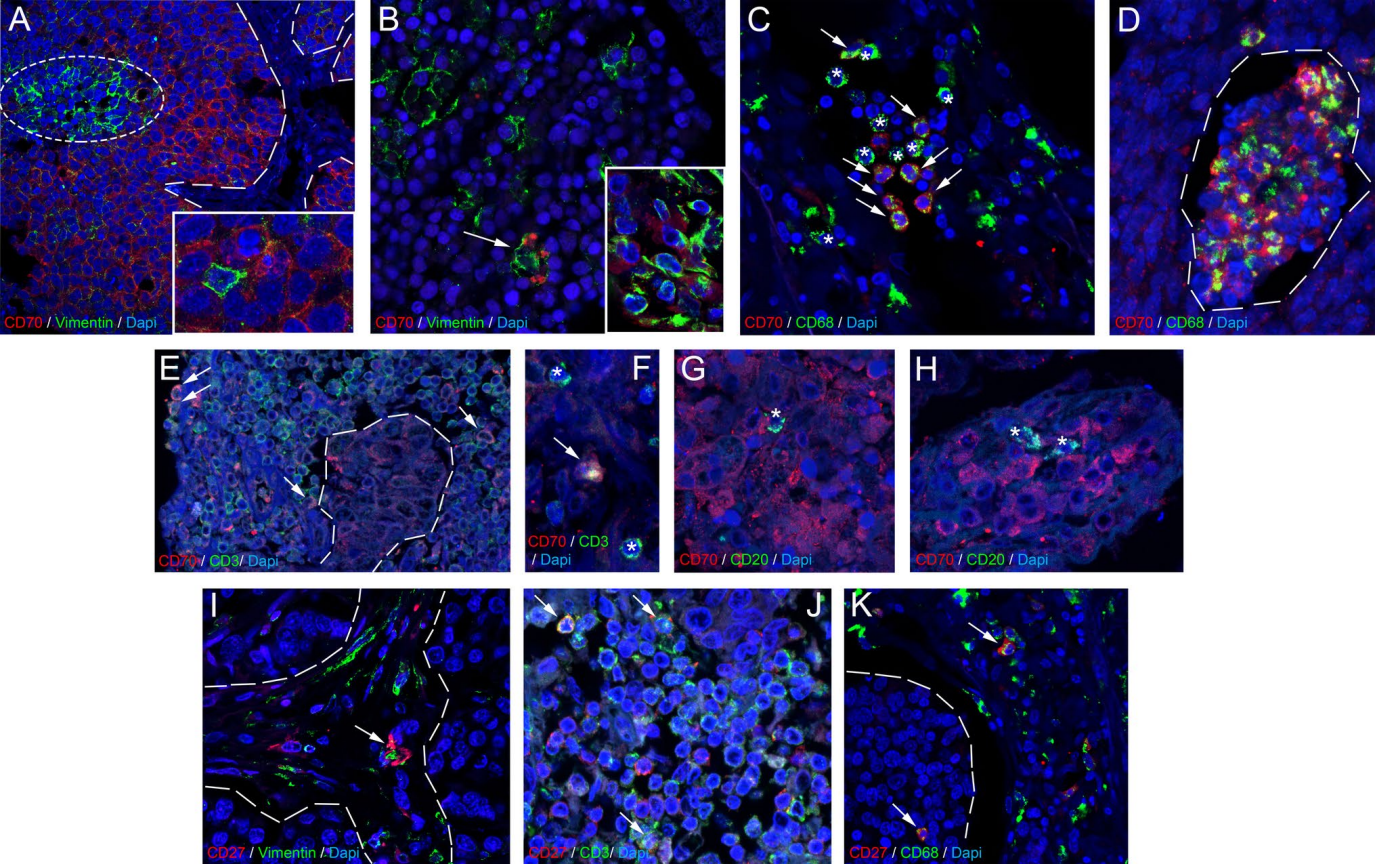
Coexpression of CD70 and CD27 on TME cells. Double IF staining revealed a diffuse CD70 protein signal in tumor nests, showing complementarity with vimentin in CD70-high patients, with lower CD70 expression in vimentin-positive tumor cells (**A**, ellipsoid area within dashed line delimited area for tumor nest). CD70 was expressed by vimentin-positive CAFs in the stroma (**B**, arrow) and CD68 + TAMs in both stroma and tumor nests (**C**, arrows, **D**). However, not all TAMs showed coexpression (**C**, asterisks). In CD70-high tumors, only a fraction of CD3 + T-cells expressed CD70 (**E**,**F**, arrows), while CD20 + B-cells showed no coexpression with CD70 (**G**,**H**, asterisks). CD27 was limited to the TME, with occasional vimentin coexpression on CAFs (**I**, arrow). Multiple CD3 + T-cells (**J**, arrows), and CD68 + TAMs (**K**, arrows) also coexpressed CD27

To reveal the qualitative expression pattern of the CD70/CD27 axis in the SCLC TME, we performed double IF stainings for EMT-marker vimentin, pan-macrophage marker CD68, pan-T-cell marker CD3, and B-cell marker CD20. Colocalization studies showed that CD70 protein exhibits a fluke, diffuse signal with a conspicuous complementarity with vimentin staining in tumor nests of CD70-high patients (Fig. 4A). Thus, vimentin-positive tumor cell clusters show lower levels of CD70-expression indicating that the epithelial phenotype is more associated with checkpoint’s expression. In contrast, CD70 was expressed by multiple vimentin-positive CAFs stroma compartment (Fig. 4B**,** arrow). Among immune cells, the majority of CD68 + TAMs seemed to express CD70 both in the stroma and the intratumoral compartments (Fig. 4C, arrows; **D**, dashed line). When observing tumor samples of CD70-high patients, CD3 staining showed that only a fraction of T-cells express the molecule (Fig. 4E,F, arrows), with most CD3 + cells being CD70-negative (Fig. 4F, asterisks). We found that even in CD70-high patient tumors, CD20 + B-cells showed no signs of coexpression with CD70 (Fig. 4G,H, asterisks). As expected, CD27 expression was limited to the TME, with no presence on tumor cells. CD27 showed coexpression with scattered vimentin + CAFs (Fig. 4I, arrow) and CD3 + T-cells (Fig. 4J, arrows) in the stroma, and with a moderate number of CD68 + TAMs in the stroma compartment and inside tumor nests (Fig. 4K, arrows).

### CD70 and CD27 expression in the clinical setting

Next, we assessed the clinical relevance of CD70 and CD27 expression in our cohort, where a total of *n* = 74 patients had eligible CD70 RNAscope score and OS data from which *n* = 51 patients were diagnosed at an early stage (I-II) and a total of *n* = 81 patients had eligible OS data and CD27 IHC score, from which *n* = 54 were in the early stage. Stage showed no conspicuous association neither with CD70 expression (*p* = 0.447), nor with CD27 expression (*p* = 0.945) according to the chi-squared test. Early-stage patients showed significantly increased OS in every setting (*p* = 0.0002), therefore, we performed all further analysis limited to this subgroup as well. Gender showed no significant association with survival (*p* = 0.112), but adjuvant chemotherapy administration showed a trend toward increased OS (*p* = 0.087). We included all three confounders in multivariate analyses.

**Fig. 5:**
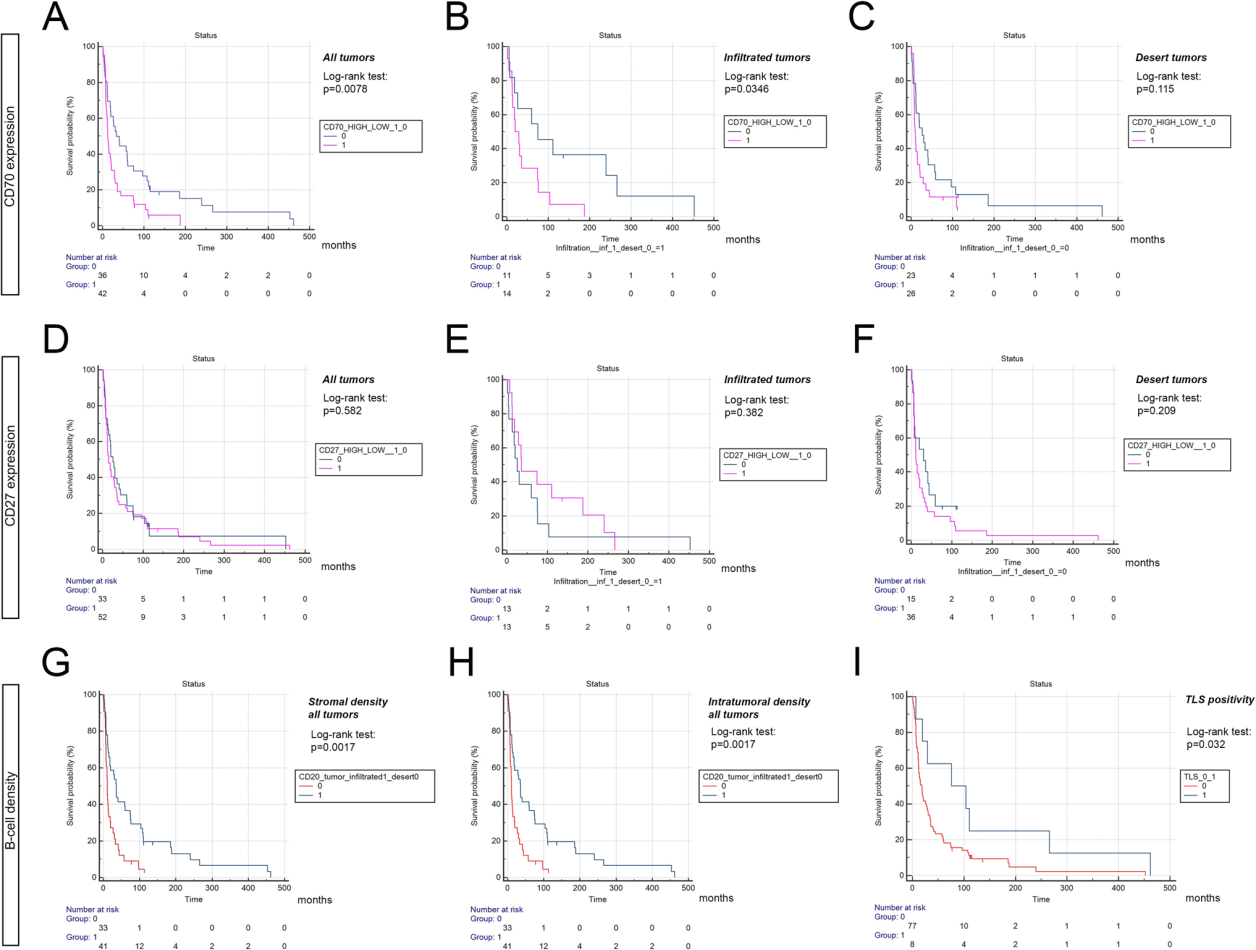
CD70 and CD27 expression and B-cell densities in the context of overall survival. CD70-high (1) patients showed significantly decreased OS compared to CD70-low (0) patients (*p* = 0.0078, HR: 1.795, **A**. CD70 was also prognostic including only patients with immune-oasis (infiltrated) tumors (*p* = 0.0346, HR: 2.21, **B**, but not in the population with immune-desert tumors (*p* = 0.115, **C**. CD27 (high (1) vs low (0)) expression did not significantly impact OS, neither in the whole cohort **D** nor in the immune-oasis (infiltrated) **E** or desert population **F**. In contrast, B-cell density was prognostic, with increased OS for patients with high (1) stromal/intratumoral B-cell infiltration (*p* = 0.0017, G,H) and TLS presence (1) (*p* = 0.0323, HR: 0.48,I)

We found that CD70-high patients exhibited significantly decreased OS (*p* = 0.0078, HR: 1.795 (95% CI, 1.127 to 2.861), Fig. 5A), and this difference remained significantly including only early-stage patients (*p* = 0.008. HR: 2.0 (1.146 to 3.49)). Interestingly, when including only immune-oasis tumors according to their CD45 + cellular densities, CD70-high patients still showed inferior OS compared to CD70-low patients (*p* = 0.0346, HR: 2.21 (95% CI, 0.972 to 5.024), Fig. 5B). In contrast, CD70 expression was not prognostic in patients with immune-desert tumors (*p* = 0.115, Fig. 5C).

Next, we assessed the prognostic role of CD27 expression, where a total of *n* = 81 patients had eligible OS data, from which *n* = 54 were in the early stage. We found no significant association between CD27 expression and OS, when classifying patients to CD27 high vs low neither in the whole cohort (*p* = 0.582, Fig. 5D), nor among immune-oasis- (*p* = 0.382, Fig. 5E), or immune-desert patients (*p* = 0.209, Fig. 5F). In the contrary, CD20 + cellular density was shown to be prognostic in SCLC, where patients with B-cell-infiltrative tumors showed significantly increased OS concerning both their stromal- and intratumoral B-cell densities (*p* = 0.0017, HR: 0.491 (95% CI, 0.292 to 0.828), respectively, Fig. 5G,H), with a significant result when assessing only early-stage patients as well (*p* = 0.0072). Also, patients with TLS present in their tumor samples showed significantly increased OS (*p* = 0.0323, HR: 0.48 (95% CI, 0.274 to 0.842), Fig. 5I).t

Cox proportional regression showed that stage (*p* = 0.0006), CD70 score (*p* = 0.0064), stromal- (*p* = 0.003) and intratumoral (*p* = 0.0024) CD20 density, and TLS presence (*p* = 0.02) is prognostic, gender (*p* = 0.098) and adjuvant chemotherapy (*p* = 0.086) is borderline prognostic, whereas age (*p* = 0.721) and CD27 score (*p* = 0.607) is not prognostic according to univariate analyses (Table 2). When including data of stromal and intratumoral CD45 + and CD8 + cellular densities from our earlier study on the same cohort (Dora et al., 13), along with all confounders below the significance threshold (p < 0.1), multivariate Cox regression showed that only stage (p < 0.0001) and CD70 score (*p* = 0.0013) remained as significant prognosticators (Table 2), indicating that in the current setting, only stage is a stronger prognostic factor than CD70 expression (Table 2).

**Table 2:** Cox proportional hazard regression

Confounder	Cox (U) *p*-value	Cox (U) Wald	Cox (U) HR	Cox (M) *p*-value	Cox (M) Wald	Cox (M) HR
Age	0.721	0.127	0.995	> 0.1		
Gender (1)	0.098	2.478	0.627	> 0.1		
Stage (1)	**0.0006**	13.012	2.564	** < 0.0001**	18.141	3.957
Adjuvant CHT (1)	0.086	2.875	0.662	> 0.1		
CD70 score	**0.0064**	7.323	1.443	**0.0013**	10.286	1.629
CD27 score	0.607	0.26	0.999	> 0.1		
stromal CD20	**0.003**	9.061	0.441	> 0.1		
tumoral CD20	**0.0024**	9.455	0.451	> 0.1		
TLS (1)	**0.02**	4.353	0.428	> 0.1		
stromal CD45	**0.0238**	5.111	0.786	> 0.1		
tumoral CD45	0.189	1.722	0.998	> 0.1		
stromal CD8	**0.038**	4.289	0.795	> 0.1		
tumoral CD8	0.106	2.607	0.955	> 0.1		

Gender (0 = male, 1 = female); Stage (0 = early stage (I-II), 1 = advanced stage (III)); Adjuvant chemotherapy (CHT, 0 = not received, 1 = received); TLS (0 = not present, 1 = present). *U* Univariate analysis with no confounders, *M* Multivariate analysis with the present confounders plus CD45 and CD8 cellular densities from (Dora et al., 13*)*

Bold indicates significant results (*p*<0.05) for Univariate and Multivariate Cox regression

## Discussion

SCLC is a highly aggressive and challenging malignancy with an alarmingly low survival rate, particularly in advanced stages where the 5-year OS rate remains below 1% [8]. Unlike NSCLC, where biomarkers such as tumor mutation burden (TMB) and PD-L1 have shown predictive value for the response to ICIs, SCLC lacks reliable biomarkers for guiding effective therapies [57]. This absence of predictive biomarkers has resulted only in incremental progress in clinical trials, with modest improvements in progression-free survival when ICIs are used ([40]; [46]). The CD70-CD27 axis, a ligand-receptor pair in the tumor necrosis factor (TNF) superfamily, plays a crucial role in immune regulation and has been implicated in various hematological malignancies. Dysregulation of this axis within the TME was also associated with tumor progression and immune evasion lately in solid cancers, making it a potential target for new therapeutic strategies in lung cancer. Preclinical study on NSCLC cell lines and tumor-bearing mice using a combination of anti-CD70 antibody and chemotherapy showed effective NK-cell mediated tumor cell killing and an enhanced intratumoral infiltration of both T and NK cells, as well as an increase in the ratio of CD8 + T cells over Tregs [15]. Furthermore, EMT promoted immune escape by inducing CD70 in NSCLC as shown by [38] and the molecule was also reported to be upregulated in EGFR mutant NSCLC cells that have undergone EMT, where drug-resistant CD70 + tumors were successfully targeted with CD70-Antibody–drug-conjugates, CAR-T Cells, and NK-CARs [36]. However, the role of the axis in small cell histologies remains unexplored.

Our findings demonstrated that CD70 and CD27 are expressed in tumor cells and within the TME of SCLC, with distinct expression patterns. CD70 expression was predominantly found within tumor nests and to a lesser extent in the stroma, while CD27 was primarily located in the stromal compartment. Notably, CD70 expression did not correlate with immune cell densities within either the stroma or tumor nests, whereas high CD27 expression was associated with reduced CD45 + and CD8 + cell densities in the stroma. There is no comprehensive expression analysis for CD70 or CD27 currently available in SCLC. Single-cell data indicates that in NSCLC, CD70 expression is mainly confined to CD4 + T-cell subsets (especially Tregs) and B-cells, with virtually no macrophage-related expression. In contrast, in SCLC, that was lately described as a cancer with a predominantly macrophage-rich TME ([12]; [13]; [32], [52]), CD70 expression has a strong representation from CD68 + TAMs, and the molecule’s coexpression on T- or B-cells is not prevalent. This is in line with our previous and currently presented data, namely that most SCLC tumors harbor a low density of T-cells [13] and B-lymphocytes. NSCLC single-cell data are more in line with our stainings on SCLC specimens concerning the expression of CD27 on T-cells, but not in B-cells, where no colocalization was detected between CD20 and CD27. Furthermore, CD27 protein is also present in TAMs, a characteristic that was not described previously in NSCLC.

In SCLC tumors, CD70 exhibited a diffuse signal with lower expression in vimentin-positive tumor cell clusters but was present in vimentin-positive CAFs in the stroma. CD70 was also expressed by the majority of CD68 + TAMs in both the stroma and tumor compartments, while CD3 + T-cells showed limited CD70 expression. CD20 + B-cells did not coexpress CD70. It was previously shown in CRC that CAFs express CD70 and CD70 + CAF density is an independent adverse prognostic marker in CRC. Functionally, CD70-positive CAFs stimulated migration and significantly increased the frequency of naturally occurring Tregs [26]. Other group came to similar conclusions where simultaneous expression of the molecule with the protein periostin on CAFs predicted worse outcomes in patients with CRC [33]. CD70 expression in GBM is known to be coupled with M2-polarized TAM densities, however, no spatial coexpression was explicitly reported ([45], [17]). The strong association of CD70 with immunosuppressive TAMs is particularly interesting, given that the CD70 expression on dendritic cells was also shown to activate anti-tumorigenic CD8 + T-cell subsets in experimental mice [30], suggesting the molecule’s context-dependent role in the TME. While multiple studies reported CD70 expression in situ on tumor cells [15], no other detailed break-down of the molecule’s TME protein expression pattern is currently available in the scientific literature. In our cohort, CD27 expression was restricted to the TME, showing coexpression with vimentin + CAFs, CD3 + T-cells, and a moderate number of CD68 + TAMs in the stroma and tumor nests. HPA data in NSCLC consistently show CD27 expression limited to the stroma with no apparent tumor cell expression (https://www.proteinatlas.org/ENSG00000139193-CD27. Apart from hematological malignancies and NSCLC, CD27 expression was detected in tumor bearing mice ([9]; RCC ([10]; [44] and nasopharyngeal carcinoma [19] among other cancers [15].

B-cells represent a TME cell type with a generally positive prognostic role [5]. Tumor-infiltrating B cells and developed TLSs have similarly been observed in lung cancer, with their phenotype differing according to the clinical stage and histological subtype [18]. Several studies have found that CD20 + B cell infiltration and the presence of TLSs are associated with a favorable prognosis ([1]; [21]; [18]; [31]; [55]) and response to anti-PD1 immunotherapy in NSCLC ([6]; [39]) making the B-cell-related immune contexture an intriguing topic in SCLC, a knowingly immune-desert tumor, with an overwhelmingly immunosuppressive TME. B-cell and TLS distribution has not yet been elucidated in any significant real-word SCLC cohort. In our cohort, both high densities of CD20 + B-cells and the apparent presence of a TLS were associated with significantly increased OS that underlines the prognostic relevance of B-cell infiltration in a previously unstudied cancer such as SCLC.

Clinically, high CD70 expression was associated with significantly decreased OS, particularly in early-stage patients and those with immune-oasis tumors. In contrast, CD27 expression did not show a significant association with OS. Multivariate analysis confirmed that CD70 expression and tumor stage were significant prognostic factors for OS, while CD27 was not. These results are in line with NSCLC, where acknowledged Kaplan–Meier (KM) Plotter tool compiling OS and RNAseq-based expression data from *n* = 504 patients from multiple cohorts [23] showed that high CD70 expression is associated with decreased OS (*p* = 0.008, HR:1.49). In contrast, the same analysis for CD27 showed a survival benefit for CD27-high patients (*p* = 0.0085, HR:0.67). In our cohort, RNAscope-base tissue expression of CD70 was shown to be an even stronger predictor of OS than CD8 + T-cell density, a basic component of the renowned Immunoscore prognostic index [5]. Moreover, when including only immune-hot SCLCs, high CD70 expression was still a significant negative prognosticator, making the biomarker intriguing for this population when administering future immunotherapies.

Limitations of this study include its retrospective, cross-sectional nature and the long recruitment period, which is explained by the low number of operable patients and tumor samples available due to disease aggressiveness. Only a limited number of patients were treated with adjuvant chemotherapy, and recruitment was before the initiation of immunotherapies, therefore we could not assess the predictive role of the CD70/CD27 axis in SCLC.

## Conclusion

Our study highlights CD70 as a key prognostic marker in SCLC. High CD70 expression, detected predominantly within tumor cells and macrophages, was linked to significantly reduced OS, also when including only early-stage or immune-oasis tumors. Notably, CD70 expression did not correlate with immune cell infiltration, indicating a distinct prognostic role independent of immune contexture. In contrast, CD27 expression was primarily found in the stroma and was not significantly associated with survival. Additionally, increased B-cell density and the presence of TLSs were correlated with improved outcomes. Given that tissue-level expression data of prognostic biomarkers are particularly valuable in this aggressive cancer, our research gives a solid basis for validation and experimental studies in the future to underpin the role of the CD70/CD27 axis in SCLC tumor immunology.

## Supplementary Information

Below is the link to the electronic supplementary material.

Supplementary file1 (JPG 4729 KB)

## Data Availability

All processed data used in this study are available in the manuscript text and supplementary material. Unprocessed data and patient metadata are available upon reasonable request from the corresponding authors.
