# Supplementary figures and images for: Expression of costimulatory molecule CD70 is prognostic in small cell lung cancer
Source: Cancer Immunol Immunother. 2025 Apr 9;74(5):165. doi: 10.1007/s00262-025-04006-2 (PMC11981989; doi:10.1007/s00262-025-04006-2)

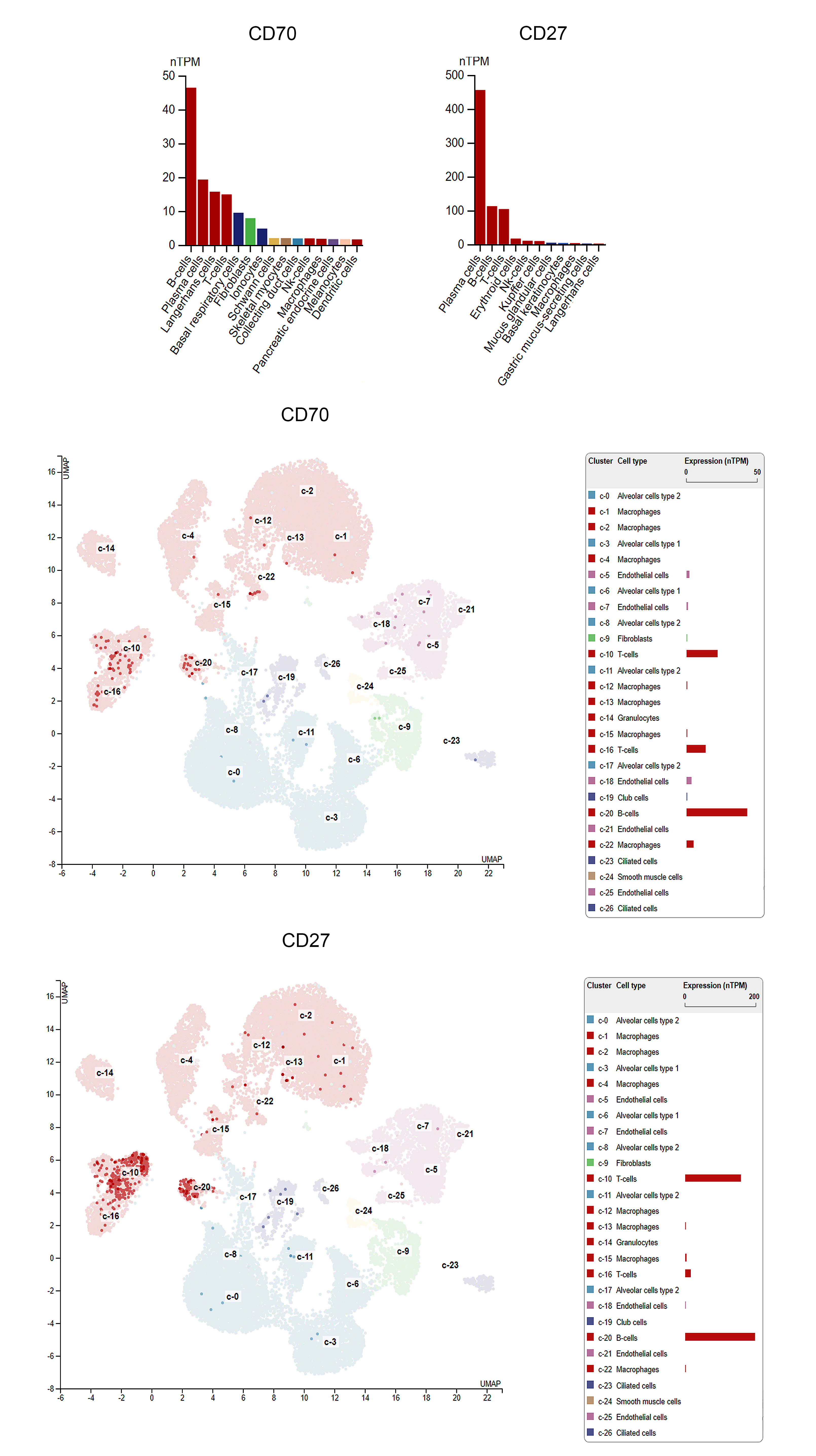

Supplement: Supplementary file 1 — Supplementary file1 (JPG 4729 KB) [file 262_2025_4006_MOESM1_ESM.jpg]
